# Acceptability of misoprostol-only medication abortion dispensed by mail-order or retail pharmacy: a qualitative study based on in-depth interviews in the United States

**DOI:** 10.1080/26410397.2025.2522567

**Published:** 2025-06-20

**Authors:** Dana M. Johnson, Sruthi Ramaswamy, Rebecca Gomperts

**Affiliations:** a Research Fellow, Department of Population Health Sciences, University of Wisconsin–Madison, Madison, WI, USA.; b Undergraduate Student, McKetta Department of Chemical Engineering, The University of Texas at Austin, Austin, TX, USA; c Founder/Director, Aid Access, Vienna, Austria

**Keywords:** Medication abortion, self-managed abortion, misoprostol, mifepristone, United States, telemedicine, pharmacy

## Abstract

The WHO recommends two medication abortion regimens, either misoprostol used with mifepristone or misoprostol-only. Both regimens are recognised as safe and effective, but in the United States (US) most abortions are completed using mifepristone and misoprostol. Given current political hostility to abortion, restrictions on mifepristone, and the long-term legal strategy to restrict nationwide access to the combined mifepristone and misoprostol regimen, more people in the US may use misoprostol-only regimens. Globally research has documented experiences with misoprostol-only abortions, but what are the experiences among people living in the US? We conducted a thematic analysis of 31 in-depth interviews with people who self-managed their abortion using misoprostol acquired from a mail-order or retail pharmacy between May and June 2020. We examine the acceptability of using misoprostol for medication abortion across three domains: the medication regimen, the mode of delivery of medications, and the overall model of care. We find that individual perceptions of misoprostol were shaped by both prior and informed knowledge. Picking up misoprostol from a retail pharmacy fostered familiarity, and having a prescription legitimised the service as an authentic medication provider. Receiving medications from the mail-order delivery model met preferences for privacy and anonymity. In reflections on the overall model, satisfaction was high across participants, but those who were adolescents at the time of their abortion had a distinct unmet need for emotional support compared to older participants. These results contribute to a growing evidence base on the acceptability of misoprostol-only regimens and mail-order and retail pharmacy service delivery models.

## Plain language summary

There are two commonly used medication abortion options. They are misoprostol used with mifepristone and misoprostol used by itself. Both regimens are safe and effective. In the United States, most medication abortions are done using misoprostol with mifepristone. Accessibility is an issue because mifepristone is legally restricted in the US and other countries. People throughout the world have been using misoprostol-only for abortions. Researchers would like to understand the experiences with misoprostol-only among people in the US. We conducted 31 interviews with US-based individuals who had an abortion at home using misoprostol. We find that knowledge shaped people's perceptions of misoprostol. Picking up the medications from a pharmacy felt familiar, and a prescription from a physician legitimised the process. Receiving medications from the mail-order order pharmacy met preferences for privacy and anonymity. Participants were generally satisfied with their abortion. Younger participants had a need for more emotional support. These findings inform the use of misoprostol-only regimens and innovations in abortion service delivery.

## Résumé

L’OMS recommande deux schémas d’avortement médicamenteux, soit le misoprostol associé à la mifépristone, soit le misoprostol seul. Ces deux schémas sont reconnus comme sûrs et efficaces, mais aux États-Unis, la plupart des avortements sont pratiqués avec de la mifépristone et du misoprostol. Compte tenu de l’hostilité politique actuelle à l’avortement, des restrictions pesant sur la mifépristone et de la stratégie juridique à long terme visant à restreindre l’accès national au schéma combiné mifépristone-misoprostol, davantage de personnes aux États-Unis pourraient recourir à des schémas à base de misoprostol seul. Les recherches menées dans le monde ont documenté les expériences avec des avortements à base de misoprostol seul, mais quelles sont les expériences des personnes vivant aux États-Unis? Nous avons réalisé une analyse thématique de 31 entretiens approfondis avec des personnes ayant pris en charge elles-mêmes leur avortement grâce au misoprostol commandé par correspondance ou acheté dans une pharmacie entre mai et juin 2020. Nous avons examiné l’acceptabilité de l’utilisation du misoprostol pour l’avortement médicamenteux dans trois domaines: le schéma thérapeutique, le mode d’administration des médicaments et le modèle global de soins. Nous avons constaté que les perceptions individuelles du misoprostol étaient façonnées par des connaissances préalables et éclairées. Se procurer du misoprostol en pharmacie a favorisé la familiarité, et la présentation d’une ordonnance a légitimisé le service en tant que prestataire de médicaments authentique. Recevoir les médicaments par le biais du modèle de commande par correspondance répondait aux préférences de confidentialité et d’anonymat. L’analyse du modèle global révèle une satisfaction élevée chez les personnes participantes, mais celles qui étaient adolescentes au moment de l’avortement présentaient un besoin de soutien psychologique non satisfait distinct par rapport à des participantes plus âgées. Ces résultats contribuent à une somme de données de plus en plus étoffée sur l’acceptabilité des schémas thérapeutiques à base de misoprostol seul et des modèles de prestation de services par correspondance et en pharmacie.

## Resumen

La OMS recomienda dos regímenes de aborto con medicamentos: misoprostol utilizado con mifepristona, o misoprostol solo. Ambos regímenes son reconocidos como seguros y eficaces, pero en Estados Unidos (EE. UU.) la mayoría de los abortos son realizados con mifepristona y misoprostol combinados. En vista de la actual hostilidad política hacia el aborto, las restricciones impuestas al uso de mifepristona y la estrategia legislativa a largo plazo de restringir a nivel nacional el acceso al régimen combinado de mifepristona y misoprostol, es posible que un mayor número de personas en EE. UU. utilicen el régimen de misoprostol solo. A nivel mundial, las investigaciones han documentado experiencias con abortos realizados con misoprostol solo, pero ¿cuáles son las experiencias de las personas que viven en EE. UU.? Realizamos un análisis temático de 31 entrevistas a profundidad con personas que autogestionaron su aborto utilizando misoprostol adquirido mediante un pedido por correo o en una farmacia minorista, entre mayo y junio de 2020. Examinamos la aceptabilidad de utilizar misoprostol para el aborto con medicamentos en tres dominios: el régimen de medicamentos, el modo de entrega de los medicamentos y el modelo general de atención. Encontramos que las percepciones individuales de misoprostol eran influenciadas tanto por conocimiento anterior como por conocimiento informado. Recoger el misoprostol en una farmacia minorista fomentaba familiaridad, y tener una receta médica legitimaba el servicio como proveedor auténtico de medicamentos. Recibir los medicamentos mediante el modelo de entrega de pedido por correo cumplía las preferencias de privacidad y anonimato. En las reflexiones sobre el modelo en general, la satisfacción fue alta entre las participantes, pero las que eran adolescentes cuando tuvieron su aborto tenían la necesidad insatisfecha específica de recibir apoyo emocional, comparadas con las participantes de edad más avanzada. Estos resultados contribuyen a la creciente base de evidencias sobre la aceptabilidad de los regímenes de misoprostol solo, y de los modelos de prestación de servicios mediante pedido por correo y en farmacias minoristas.

## Introduction

The World Health Organization (WHO) recommends two regimens as safe and effective for medication abortion: misoprostol used with mifepristone and misoprostol used alone (commonly referred to as misoprostol-only).^1^ Throughout the world, misoprostol-only regimens are commonly used because misoprostol is relatively low cost, straightforward to use and shelf-stable. Misoprostol also has several applications beyond medication abortion, including the prevention and treatment of stomach ulcers, postpartum haemorrhage, miscarriage management and labour induction. Due to this wide range of uses, misoprostol is often available in pharmacies, clinics and community distribution settings throughout the world. This utility and availability make misoprostol a valuable medication,^2^ especially in settings where mifepristone is not available and/or where abortion is illegal or restricted.^3^ Misoprostol-only regimens have expanded access to safe abortion throughout the world and are linked to reductions in unsafe abortion-related maternal mortality in Africa, Asia and Latin America.^4,5^

Research in Latin America,^6^ Asia,^7,8^ Africa^9^ and the United States (US)^10^ evaluating the safety and effectiveness of misoprostol-only medication abortion finds high rates of effectiveness and few incidents of serious adverse events. Yet in contrast to global abortion provision norms, US providers typically prescribe mifepristone with misoprostol. In 2000, the US Food and Drug Administration (FDA) approved the mifepristone and misoprostol medication abortion regimen in the US. Mifepristone blocks the absorption of progesterone, so the lining of the uterus begins to shed. Then 24 hours later, misoprostol is taken, causing the uterus to contract and expel the pregnancy. FDA approval of the combined mifepristone and misoprostol regimen came after decades of advocacy from researchers and activists,^11^ and upon approval this regimen became the standard for clinicians in the US.

Today, this poses a major access problem because mifepristone is substantially restricted in the US. In June of 2022, the US Supreme Court decision in *Dobbs vs. Jackson Women’s Health Organization (Dobbs)*, overturned the 1973 *Roe vs. Wade* ruling that legalised abortion in the US.^12^ Following the *Dobbs* ruling, at least 27 states have enacted abortion bans or substantial restrictions,^13^ making the provision of mifepristone heavily restricted or impossible in these states. Prior to the *Dobbs* decision, access to mifepristone was curbed by the Risk Evaluation and Mitigation Strategies (REMS) program, which restricted the dispensing of mifepristone. In 2020 and 2021, temporary adjustments to mifepristone’s REMS classification allowed the medication to be dispensed by mail, allowing for telehealth provision of mifepristone. The in-person dispensing requirements were permanently removed in 2023.^14^ However, access to mifepristone was threatened by the 2024 US Supreme Court Case *Alliance for Hippocratic Medicine vs. US Food and Drug Administration (FDA)* which sought to remove the FDA approval of mifepristone, potentially forcing the drug off the US market. Although this case was initially unsuccessful,^15^ in October 2024 US state Attorneys General from Missouri, Kansas and Idaho filed a joint amended lawsuit renewing efforts to ban mifepristone.^16^ These prior restrictions and current challenges are part of a long-term legal strategy to restrict nationwide access to the combined mifepristone and misoprostol regimen.

Due to this highly restrictive policy context and potential future restrictions on mifepristone, researchers suggest that misoprostol-only medication abortion regimens warrant renewed attention,^17^ and US providers have updated clinical practice guidelines to include misoprostol-only regimens.^18^ Yet, there is still only a small body of US-based research focusing on misoprostol. Prior clinical research confirms the safety and effectiveness of misoprostol-only,^19^ and qualitative research finds that prior reproductive experiences informed participant’s knowledge, preparedness, pain management, and ability to both recognise and manage potential (although rare) complications while using misoprostol.^20^ Considering this evidence base, little is known about the acceptability of misoprostol-only medication abortion among people living in the US. Understanding the acceptability of misoprostol-only regimens is key as people in the US may have different perceptions of misoprostol regimens compared to the mifepristone and misoprostol regimen. Understanding the acceptability of in-person and mail-order acquisition of medications is important as service delivery innovations continue to develop, and telehealth provision of medication abortion is rapidly expanding in the US.^21^ Furthermore, understanding the acceptability of any abortion care model (including regimen and mode of delivery) is critical for informing person-centred innovations in abortion care provision and ensuring reproductive autonomy, the fundamental human rights concept of empowering individuals to decide about and control the matters associated with pregnancy and childbearing.^22^

To address this research gap, we use in-depth interviews and thematic analysis to examine acceptability across three domains: the medication abortion regimen, the mode of delivery of medications and the overall model of care.

### Acceptability of health care interventions

In healthcare, acceptability is a “multifaceted construct reflecting the extent to which people delivering or receiving a healthcare intervention consider it to be appropriate, based on anticipated or experienced cognitive and emotional response to the intervention”.^23^ Acceptability is also a central part of access, including the cultural and social factors determining the possibility for people to accept aspects of a health service, and the determined appropriate modalities of delivering care.^24^

**Figure 1.**
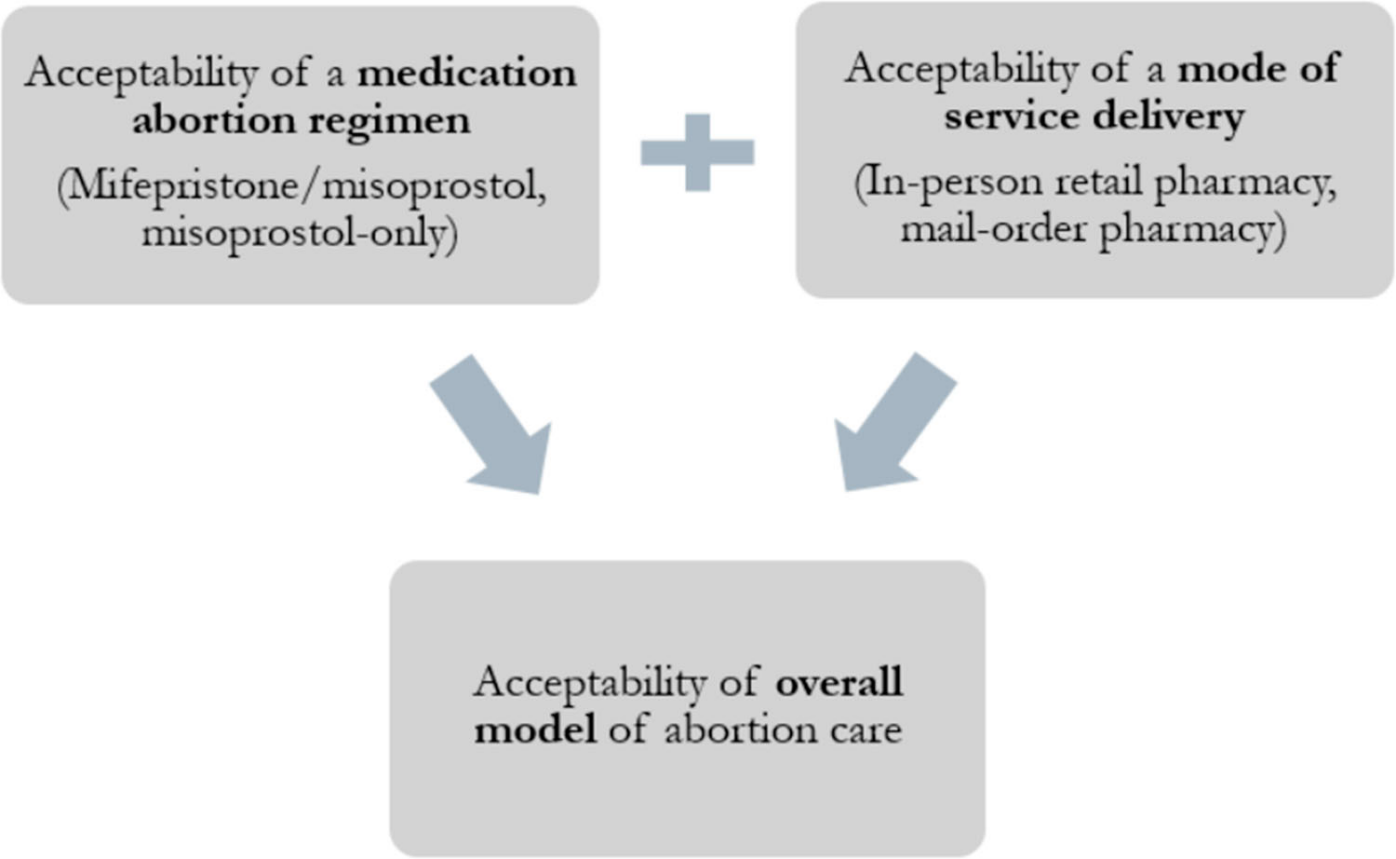
Tailored conceptual model of acceptability examined across three domains that encompass the process of medication abortion using misoprostol dispensed from a mail-order or retail pharmacy

To understand acceptability, we use Sekhon et al.'s^23^ acceptability framework, with a theoretical focus on the retrospective acceptability of individual perceptions, experiences and satisfaction.^23^ This framework informs our tailored conceptual model (Figure 1) of acceptability examined across three domains that encompass the process of medication abortion using misoprostol dispensed from a mail-order or retail pharmacy. First, **medication abortion regimen:** what are the individual perceptions of misoprostol-only? Second, **mode of abortion service delivery:** what are individual experiences with delivery of misoprostol by mail-order pharmacy? Third, **overall model of abortion care:** what were individual reflections on the abortion process as a whole? What are individual perceptions among participants, as well as reflections on overall satisfaction?

Perceptions of misoprostol-only compared to mifepristone and misoprostol, and the physical experiences with using misoprostol-only are published elsewhere.^19^

## Methods

### Sample and data collection

We conducted 31 anonymous, semi-structured, in-depth interviews with individuals who in May and June of 2020 acquired misoprostol from a non-profit online telemedicine service. Since 2018 this service has provided medication abortion pills and instructions for self-managed medication abortion (obtaining and using medication abortion outside the formal health setting, and without the direct supervision of a clinician) to people in all 50 US states. Typically, the service prescribes mifepristone and misoprostol in line with the American College of Obstetrics and Gynecology guidelines for medication abortion provision.^14^ Due to difficulties mailing mifepristone internationally during the COVID-19 global emergency, the organisation temporarily adjusted their model to prescribe misoprostol-only distributed by pharmacies in the US.

Misoprostol was available to people up to 10 week’s gestation. People seeking abortion completed an online consultation comprising a series of questions about reproductive and general health history, which were reviewed by a physician to determine eligibility for medication abortion. If eligible for treatment, a clinician prescribed three doses of 800 micrograms of misoprostol, either mailed directly through a mail-order pharmacy or available for pick-up at a brick-and-mortar retail pharmacy. Detailed instructions were provided via email for using the 800 micrograms of misoprostol sublingually every three hours for three doses. The clinician prescribed an additional 800 microgram dose of misoprostol if expulsion did not occur after several days. A sliding scale suggested an optional donation of $35, and medications were available at no cost for those who could not afford the donation. Some participants also paid additional co-pays at their pharmacies, which ranged from $5 to $25.

Interview participants were recruited by an email invitation sent by the service on 1 November 2021. This email invited people to participate in an in-depth interview with researchers at the University of Texas. Potential participants were informed they would be compensated $50, and results would be used to inform research and policy to expand access to abortion. Participants were asked to download the encrypted messaging application signal and send a text to schedule a conversation with the study team. To be eligible to participate, individuals had to be at least 18 years old at the time of the interview. Verbal informed consent was obtained at the beginning of each interview.

Interviews were conducted between 5 November 2021 and 13 December 2021. All interviews were conducted by phone only, in English, using the messaging application that ensured participant anonymity. After participants contacted the research team, they were immediately assigned pseudonyms, and no identifying information was collected. With the permission of participants, interviews were audio recorded, and recordings were reviewed internally to check for any potential identifying information. Interviews lasted between 40 and 90 minutes, and participants received a $50 digital gift card to a major commercial retailer.

Interviews were conducted by two members of the research team using a semi-structured in-depth interview guide designed to capture experiences with and perceptions of self-managed medication abortion using misoprostol. We took detailed field notes following each interview, which were discussed by the research team as patterns and themes emerged throughout data collection. The final sample size of 31 interviews reflected the research team’s determination that a balance between thematic saturation and available resources had been reached. The study was approved on 22 October 2021, by the Institutional Review Board at the University of Texas at Austin (Ref. 2018080061). We used the Standards for Reporting Qualitative Research (SRQR) research guidelines.^25^

### Analysis

Interviews were transcribed verbatim using a University of Texas at Austin transcription service. Two members of the research team co-developed the initial coding scheme based on our research questions, prior literature and theoretical framework of acceptability. We discussed and refined the final coding guide with input from two external self-managed abortion content experts. We coded transcripts with a flexible coding^26^ approach using ATLAS.ti 7 (Atlas.ti) software. We established intercoder consistency by coding the first three transcripts together and then comparing codes.^27^ We discussed these three transcripts and determined how to reconcile code discrepancies. We then refined the coding guide a second time and completed coding the remaining transcripts. Coding was considered complete when no new codes emerged, and all code notes had been reconciled or extensively discussed.

Guided by a focus on the core category of experiences with misoprostol, the lead author conducted a second round of axial coding concentrating on the subcategories of knowledge of misoprostol, perceptions of misoprostol and experiences with the two pharmacy acquisition models. Throughout the coding and analysis process, the first and second authors compared emerging themes,^28^ engaged in detailed memo writing, reflected on interview field notes and had a weekly discussion of thematic findings. Throughout the interview, coding and analysis process, the two members of the research team who conducted interviews and analyses reflected on their roles and potential biases. Self-managed abortion is a stigmatised and criminalised health behaviour,^29^ and despite these realities participants were open to discussing their experiences. We believe that this is because our study design may have legitimised us as trustworthy people to speak to. We recruited directly from Aid Access, with the support of staff, and the founder/director was a member of the study team. Prior to interviews, we asked potential participants to download an encrypted application and ensured them that we will not collect identifying information. This commitment to confidentiality throughout recruitment and interviews may have helped establish trust, and a baseline acknowledgement of the political context surrounding the conversations. Furthermore, the timing of the interviews is worth noting. Interviews were conducted in November 2021, two months after the state of Texas enacted Senate Bill 8, the early gestation abortion ban passed by the Texas legislature. At the time, this was the strictest abortion ban in the country, and it received widespread media coverage. Participants frequently referenced the “Texas abortion ban”, and because we self-identified as researchers at the University of Texas, these discussions established rapport and a sense of solidarity between researchers and participants

## Results

### Sample characteristics

Thirty-one people participated in interviews, and participant sociodemographic (Table 1) and geographic characteristics (Table 2) were gathered at the conclusion of interviews. At the time of the abortion, participants were aged 17–44 years, and most (*n* = 22) were aged 25–39 years. Participants were racially diverse, as most (*n* = 16) identified as a race other than white. Most (*n* = 23) were working full time and had health insurance (*n* = 28). In terms of geography, the majority lived in a state classified in 2020^30^ as hostile to abortion rights (*n* = 18) and most lived in a city or suburb (*n* = 24). Eleven participants received misoprostol from the mail-order pharmacy, and 20 picked up misoprostol at a retail pharmacy.

**Table 1.** Self-identified characteristics of individuals who used misoprostol alone used for self-managed medication abortion in the United States between May and June of 2020 (*N* = 31)

Characteristics	Frequency *n* (%)
**Age**	
<18	3 (10%)
18–19	1 (3%)
20–24	4 (13%)
25–29	7 (23%)
30–34	6 (19%)
35–39	9 (29%)
40–44	1 (3%)
**Race/Ethnicity**	
Asian	1 (3%)
Black/African American	6 (19%)
Hispanic/Latinx	6 (19%)
Native American	1 (3%)
Native & Hispanic	1 (3%)
West Indian	1 (3%)
White	15 (50%)
**Gender**	
Female or Woman	31 (100%)
Other	0 (0%)
**Sexual Identity**	
Asexual	1 (3%)
Bisexual	6 (19%)
Heterosexual	20 (65%)
“I like men”	1 (3%)
Pansexual	1 (3%)
“Lesbian”	1 (3%)
Missing	1 (3%)
**Number of children at the time of their abortion**	
0	16 (52%)
1+	15 (48%)
**Highest level of education at the time of their abortion**	
High School	5 (16%)
Associates	3 (10%)
Some college	13 (42%)
Bachelor’s Degree	5 (16%)
Graduate Degree	5 (16%)
**Employment at the time of their abortion**	
Working full time	23 (75%)
Working part time	1 (3%)
In school/Student	2 (6%)
Working and in school	1 (3%)
Full-time caregiver	4 (13%)
**Insurance at the time of their abortion**	
Affordable Care Act	1 (3%)
Employer	14 (45%)
Medicaid	6 (19%)
“Public insurance”	1 (3%)
Parent/Guardian	2 (6%)
Partner/Spouse	2 (6%)
Uninsured	3 (10%)
Veterans Affairs	1 (3%)
Missing	1 (3%)

**Table 2.** Geographic characteristics of individuals who used misoprostol alone used for self-managed medication abortion in the United States between May and June of 2020 (*N* = 31)

Geographic characteristic	Frequency *n* (%)
**2020 State Policy Context**	
Very hostile	5 (16%)
Hostile/Leans hostile	18 (58%)
Middle ground	1 (3%)
Supportive/Leans supportive	4 (13%)
Very supportive	2 (6%)
Declined	1 (3%)
**Geographic area**	
City	13 (42%)
Suburb	11 (35%)
Town	4 (13%)
Rural area	3 (10%)

### Thematic findings

Four themes emerged: (1) individual perceptions of the medication abortion regimen were shaped by prior and informed knowledge of misoprostol. (2) Individual experiences with the retail pharmacy pick-up model of service delivery were largely positive. (3) Individual experiences with the mail-order pharmacy delivery model met preferences for privacy and anonymity. (4) Participants were grateful for access to abortion care and described satisfaction with the overall model of abortion care.

#### Medication abortion regimen: Perceptions of misoprostol were shaped by prior knowledge

Eighteen of the 31 participants discussed perceptions of misoprostol shaped by prior knowledge*.* At the time of request, people were notified by the service of the process and effectiveness of the medication. The service notified people that “Misoprostol is a medicine that can cause an abortion in 94% of cases. It is less effective than the combined Mifepristone and Misoprostol”. This inevitably shaped perceptions of the misoprostol, and in interviews participants frequently raised the effectiveness rates of misoprostol-only regimens. Participants also explicitly compared misoprostol-only and mifepristone and misoprostol effectiveness rates, at times quoting the scientific literature. Yet what was prominent throughout interviews was that prior knowledge shaped perceptions of misoprostol-only regimens. For Marcia,* a 31-year-old Hispanic woman living in the Southeast, misoprostol was familiar to her from her experience growing up in the Dominican Republic. As she put it:

*“Where I'm from, ladies do it all the time. It's just here that it's so hard to get the medicine and the information, which is, I don't know, so weird because I'm from a third world country and this is a first world country. But in my country, ladies do it all the time, in their house, safely. So, I know about it. The hard part was to get the medicine. That was the difficult thing. Yeah. That's a big deal here.”*

Marcia’s experiences normalised misoprostol’s use for abortion care, highlighting the fact that in other countries, misoprostol is known as the typical regimen.

Some participants held similar prior knowledge from using misoprostol during other reproductive experiences. As Sammy, a 24-year-old white woman explained:

*“Yeah, I had had multiple abortions before that. All of them, except for one - I’ve had at least 5 - all of them except for one were the pills. When I went to the clinic, to do those, it was only like a total of 4 pills. But with [the service], it was like 12, I want to say? Yeah, it was three doses of four pills under the tongue.”*

Carolyn, a 34-year-old white woman said: “*The only usage I know of it is, you can use it to induce people, you can use it to get the rest of miscarried products out. That was my experience with it*”.

And Eleanor, a 20-year-old white woman discussed misoprostol’s use for menstrual regulation:

*“I knew like what it [misoprostol] did but I didn’t know you could like use it for that, I know they have something similar to it where if you don’t have your period so long, you can automatically shed the lining of your uterus, I knew that was a thing, but I didn’t know you could also do it like that.”*

Other participants held prior knowledge from their professional work as nurses, doulas, or in veterinary care. Shelly, a 29-year-old Black woman, held a combination of the two. When she first read news articles about ordering abortion pills from a website, she wanted to confirm that this was also the medication she had used for an in-clinic abortion years ago, explaining:

*“I did my research on it and by the way … I have a master’s in health administration and education. I’m a health educator. I worked with the state … so I did all my research. Like I knew that this was the same medication.”*

#### Medication abortion regimen: Perceptions of misoprostol were shaped by informed knowledge

Twenty five of the 31 participants discussed perceptions of misoprostol shaped by informed knowledge of misoprostol, informed by internet searches, news articles, Reddit and Facebook forums, and their personal networks. Through informed knowledge, some participants, particularly younger participants, were learning about medication abortion for the first time. As Danielle, a 17-year-old Asian-American woman explained:

*“I had realized that I mean, initially I didn't even know a pill existed. I always believed it was a D&C procedure that everyone demonizes and the gruesomeness of it. But there's a pill available and I had begun looking into that pretty extensively seeing what options I could take to find it. And [the service] was something that popped up. And I looked into it and was able to get the medicine that I needed … ”*

And Kristine, a 17-year-old white and Hispanic/Mexican woman said:

*“I didn't know how it worked until I searched online and did, like, a bunch of research and, you know, I figured out there were pills. I figured out so many things that, like, I didn't know about it.”*

Others discussed how learning about medication abortion, including the combined mifepristone and misoprostol regimen shaped their perception of misoprostol-only. Leigh, a 17-year-old Black woman, was motivated to learn more about the safety of misoprostol after learning about the medication from the service. Leigh was searching for abortion care after she was sexually assaulted, all while managing her parents’ divorce and the daily demands of high school. The safety and effectiveness information she found herself while searching online and reading Reddit forums provided her a sense of control over a situation that she described as incredibly isolating. However, learning different regimens also shaped her perception of just using misoprostol:

*“There’s a lot of research about abortion in general. [I] did a lot of research about the medication that [the service] was able to provide me, which was misoprostol. But realized soon down the line that that's not the only medication that you need in order to go through a medicated abortion.”*

Conversely, Valerie, a 40-year-old white woman, read about the combined regimen and was satisfied with having the misoprostol option, explaining: “*From what I had read up on the study said that it [mifepristone] wasn't needed, only in later term pregnancies, and I knew mine was very early*”.

For those acquainted with misoprostol from previous experiences, recalling this knowledge provided consolation when navigating the new process of using an online telemedicine organisation to order medication. Among those who were learning about misoprostol for the first time, they were motivated to find accurate information about the medication.

#### Mode of service delivery: experiences with the retail pharmacy pick-up model

Participants were familiar with and reassured by the retail pharmacy pick-up model, reflecting comfort and familiarity with pharmacies as an institution of medication and healthcare acquisition. Some had initial concerns about interacting with pharmacists, but overall, there were few instances of negative experiences with pharmacists.

Twenty of the 31 participants discussed experiences picking up misoprostol from a local brick-and-mortar retail pharmacy. Some participants were initially unsure about the legitimacy of the online telemedicine service, as Leah, a 29-year-old Black woman in the South said: “*I was kind of skeptical at first. It was like kind of sketchy*”*.* But a pharmacy prescription confirmed that the website was an authentic medication provider.

Sammy, a 24-year-old white woman, was in the process of leaving an abusive relationship and caring for her young daughter. When reflecting on her abortion, she said: “*[the service] saved my life*”*.* As she progressed in her pregnancy timing was tight and she needed the abortion soon. She felt she may be taking a risk by ordering pills from a website, but as soon as the physician sent the prescription to her local CVS, she felt at ease:

*“I had talked to an actual doctor – and I can’t remember – I think I had only talked through email, but I felt okay once CVS took the prescription. There’s no way CVS would give this to me if it was a scam. So that’s really how I started to trust it.”*

Most people felt familiar with the pharmacy as an institution that provided health services, and familiarity with pharmacy location, hours and staff, eased some stress associated with abortion care. Some made explicit comparisons to the stress they’ve felt at clinics, walking past protestors and waiting for hours in the lobby. Overall, people were relieved to be simply picking up the medication to use privately at home. Jessica, a 27-year-old Native and Hispanic woman from the Midwest said that the retail pharmacy model felt like it was “*almost too easy … like … why haven’t we been doing this the whole time?*” She was thrilled that she could access pills privately, saying: “*I was grateful, very grateful, that something like this was available*”*.* Similarly, Shae, a 27-year-old white woman recalled: “*It was super easy. I just went through the drive-thru and they even took my insurance for [the medication], so I didn't have to pay full price, so it was pretty simple*”.

Others felt worried that due to the sensitive nature of abortion, the pharmacist might ask intrusive questions, refuse to fill their prescription, or disclose private information about their pharmacy visit to others. Danielle explained:

*“I had moved to a very conservative state during Covid. And I remember just walking into CVS and thinking, are they going to give me the prescription? But the entire experience at CVS was very simplistic. I went in, I got my medication and despite sweating bullets and being so extremely nervous, everything worked out.”*

Brenda, a 38-year-old white woman was initially concerned because “*Some pharmacists have a hard-time filling prescriptions from doctors they’ve never heard of*”. To prepare for any push-back, Brenda memorised a script explaining why she needed the medication. When she saw that the pharmacist was female, she was relieved:

*“I very much so lucked out that the pharmacist that was on that week was female … she didn’t ask a lot of questions. She just verified that it was what I wanted to do, and she filled the prescription right away.”*

Brenda chose her usual pharmacy because she preferred knowing the pharmacists and other staff, but two participants asked the organisation to send the prescription to a pharmacy that was not their typical location, fearing that a staff member would disclose that they were picking up abortion pills.

Overall, despite concerns, reported instances of pharmacist push-back or refusal were rare. Only one participant had a pharmacist outright refuse to fill the misoprostol prescription. According to Marcia: “*The lady was a little rude and she’s like ‘No, I cannot give you that. That’s too many’ or whatever. And I was like, okay. I just walked away … I was a little scared, so I changed pharmacies*”. Marcia relayed the event to the service, and they immediately sent her prescription to a second pharmacy where “*She explained it to me, no judging. She just told me what I need to do*”.

Another participant was contacted by the pharmacist after she picked up the misoprostol. Trish was leaving the pharmacy when she received a phone call:

*“I was still in the parking lot and she told me, she’s like, ‘Don’t even touch it. You can bring it back and I can dispose of it.’ And I was like ‘No, it’s fine.’ I didn’t really know what to say. I was so caught off guard. And I don't even remember how I ended the conversation, but I went home because I knew what I was getting it for.”*

She was rattled by this encounter, but did proceed with taking the misoprostol, explaining: “*It’s not your pharmacist’s choice … it should be your choice and only your choice*”*.* Although some participants were worried, the majority had smooth experiences. The familiarity of the pharmacy legitimised the new processes of requesting misoprostol from an online service.

#### Mode of service delivery: experiences with the mail-order pharmacy model

Ten of the 31 participants lived in states where they could receive misoprostol delivered to them by mail. Complementing concerns about in-person pharmacists, the mail-order pharmacy delivery model met preferences for privacy and anonymity. Elaina, a 20-year-old white woman from the South explained:

*“[I] loved it, I didn’t even have to go anywhere, like nowadays when you go to a pharmacy, you don’t even get to grab your medicine and go, you have to like sit there and have a consultation with a doctor, and like, I didn’t want that, them to be like ‘Have you used this medication before?’ and I’m like ‘No’ and them be like ‘Well what are you getting it for?’ and I can’t imagine then saying like ‘Hey I’m having an abortion,’ right?”*

Leah was worried that her abusive partner would find out about the pregnancy, and she did not want to explain to anyone why she was leaving the house during COVID-19 and driving to a pharmacy. She explained that:

*“I just think it was easier for me to just have it delivered so I wouldn't have to leave the house to go to a Walgreens or CVS and kind of get the medication … I did like, I guess, the privacy part of not having to go to the actual pharmacy … at that time of being vulnerable, I just wanted it to be at home, just delivered to me. Just open my door, get my pills, and take it.”*

#### Overall model of abortion care

When reflecting on the overall model of abortion care, participants primarily discussed gratitude for an abortion care option when other options (especially abortion care at a clinic) were limited or impossible. Embedded in discussions of gratitude for care, were both unmet needs for emotional support among a subgroup of adolescent participants and satisfaction with the supportive features of the service.

##### Distinct unmet need for emotional support among adolescent participants

Three of the 31 participants were adolescents under 18 at the time of their abortion, and their reflections on the overall model of care presented a departure from older participants. Their reflections illuminated a lack of support in the abortion process, yet gratitude for this abortion care option. When Kristine found out she was pregnant she was in the process of becoming emancipated and moving across the country. She immediately turned to the internet for information and found the service’s website “while Googling”. The clinic felt off limits because of the shame she felt about needing an abortion, and the perceived stigma surrounding teenage pregnancy and abortion made her entire experience very isolating. As she explained:

*“I wish the world was different, society was different. People were more understanding and accepting, especially in regards to friends and family. If I had someone with me, if my partner who had contributed to the pregnancy was helpful, it would have made a world of difference to not feel so alone and so scared and frightened.”*

Danielle described feeling grateful for this abortion care option. In 2020, she ordered misoprostol from the service (the focus of the interview), at the conclusion of the conversation, she mentioned that she was so satisfied with her experience that when she needed abortion care again in 2021, she ordered pills from this service again. For both abortions, she felt like the service and this option was “*the only thing that was there for her*”, noting that “*it’s scary to know that not everyone has resources and the opportunities to have a choice like I did*”.

When Kristine found out she was pregnant she explained:

*“I had a lot going on. I had just kind of moved schools, ’cause I was sexually assaulted by a classmate and I was having a hard time with my boyfriend at the time. We weren't doing too good, and you know, then I found out, and he wasn't very happy about it, and neither was I. I mean, I didn't wanna keep it. I just had too much going on emotionally.”*

At the time she was working through her panic disorder and experiencing “a lot of anxiety”. The clinic did not seem like an option because even though she knew her state of residence did not require parental consent to obtain abortion services from a clinic: “*just going out in public places … I’d get like, panic attacks, like leaving my house for too long*”*.* When she found out she was pregnant, she explained:

*“… it was hard emotionally, just cause of everything that was going on and I didn't feel like I had any support. I didn't feel like there's anyone there or anyone who understood why I would make that decision.”*

These participants faced specific barriers to the clinic and were navigating tumultuous life events, including abortion and pregnancy stigma, emancipation and sexual assault. Like older participants, they had knowledge about medication abortion and misoprostol. Yet the loneliness they articulated and the intensity of an expressed need for emotional support was not reflected in the broader sample.

##### Satisfaction with the supportive features of the service

Among older participants, gratitude for this abortion care option was also a consistent theme. Taking misoprostol was described as physically taxing and scary for some, and a lack of external support for abortion presented emotional challenges. Participants discussed gratitude for the comfort and care the overall model brought, especially supportive features of the service and the fact that when they needed abortion care, this was one of the few places that provided instructions, authentic medication, and support for abortion.

Brenda was living out West and searching for abortion care after being sexually assaulted. Her experience with misoprostol included intense cramping and she was working through the complex emotions associated with the pregnancy and her decision to have an abortion. Yet when she reflected on her overall experience with the service, she was clear: “*They were perfect. They were absolutely what I needed when I needed them*”.

Carlee was a 30-year-old Black woman living in the Southeast. She had just moved, started a new job, and was “*getting back into the workforce*”*.* When reflecting on why she sought abortion care, she said that it was “*not the right time to have a baby*”*.* Her experience with misoprostol echoed similar themes to Brenda’s experience: “* … it was smooth … of course it was emotional … but it was a good experience*”.

Trish was a working mother of 5. As the sole breadwinner for her family, she couldn’t take off work for multiple appointments at an abortion clinic. She was relieved when she found out she could pick-up misoprostol from a pharmacy and her Medicaid covered the pharmacy co-pay. She said:

*“They did everything that I could have possibly asked for. I mean, they sent me my medication, they didn't ask me for a dime, they checked on me, they were basically holding my hand through the whole thing from over my email, but they were there the whole process. So I'm very grateful for the experience I had with them.”*

Others reflected that if they needed to have an abortion again, they would use the service, and two participants had used the service more than once. Jade, a 26-year-old white woman explained:

*“If I did have to go through it again then I would definitely go through [the service] again … the whole process, every part of it was in a way where I was like “yeah, I would do it again”.”*

Carlee had similar thoughts, saying:

*“I would do it all over again. It was … It was like no hassle. And it was like confidential, you didn’t have to leave your house, well you did to go pick it up but its everything was confidential and private.”*

And Kristine explained that the process:

*“Just, just the whole thing, the whole experience … the communication, the emails, the instructions the information, and receiving the medications and then taking them. It was all pretty much, like, pretty easy.”*

Participants who did not have a complete abortion or had a complication while using misoprostol also expressed elements of satisfaction. Shelly had a dilation and curettage procedure for incomplete abortion, yet she still felt the model worked well:

*“[The service] has worked really well for me. Being able - the, the ease of you know, doing, doing everything online, doing everything virtually, having the prescription mailed to me, being able to, you know, go through my abortion process at home, in the comfort of my own home. And it was all cost efficient.”*

Melanie, a 38-year-old white woman, also had a dilation and curettage procedure for incomplete abortion. Yet overall, she appreciated the supportive features of the abortion care model, despite her experience with misoprostol-only and an expressed preference of the mifepristone and misoprostol regimen:

*“They can't help how my body handled the medication. Really, chemical abortion is a two-step process. So, they did the best that they could, and they offered what they could, which works for a lot of women, but it didn't work for me. So, I would have to say [the service] did because that gave me comfort, just knowing that, ‘Okay, I've made this decision.’ I know it's going to come in the mail. I know I have support with my best friends, and I can reach out and ask questions, or whatever … I'm very grateful that [the service] was there.”*

When asked if they would use this service again, Melanie said she would not, because “it didn’t work for me”, yet Shelly felt: “Oh yeah, for sure. I mean, if I was, if I was faced with that situation again, I would 100% go through [the service] again”.

## Discussion

In this study, participants held a range of perceptions of misoprostol and experiences with service delivery, reflecting the diversity of experiences people have with misoprostol, and medication abortion generally. Overall, participants found the regimen and two service delivery models to be acceptable, primarily because of the supportive features of the model and the delivery of abortion care when access to the clinic was restricted or impossible. However, participants discussed unmet need for emotional support and access to abortion broadly, highlighting that although this was a critical option when people needed care, that restrictions on all medication abortion methods and models impact individual wellbeing.

Given current political hostility to abortion and increasing restrictions on mifepristone, more people in the US may use misoprostol-only regimens, and the acquisition of abortion medications from in-person and online pharmacies is rapidly growing in the US. These results contribute to a growing evidence base on abortion acceptability in several ways. First, these results are in line with global research finding misoprostol-only to be an acceptable medication abortion regimen,^31–33^ and fill a gap in the US-based medication abortion acceptability literature which largely focuses on mifepristone and misoprostol regimens. Second, findings are aligned with the literature on the acceptability of mail-order pharmacies (or telemedicine). We find that mail-order dispensing offered comfort and privacy, consistent with other studies of self-managed medication abortion acquired by mail^34^ and telehealth medication abortion in the formal health care setting.^35,36^ This is promising, as post-*Dobbs* researchers find a marked rise in demand for telehealth mail-order dispensing medication abortion, and the rapid expansion of mail-order dispensing to people in restrictive states by providers operating under protective Shield Laws.^37,38^ Third, findings on the acceptability of pharmacy pick-up are consistent with prior research finding patient satisfaction with pharmacy dispensing of mifepristone and preference for medication abortion availability through prescriptions from primary care clinics with medications dispensed in pharmacies.^39^ However, the literature to date focuses on acceptability of the modality and has yet to parse out individual experiences with the pharmacy site and staff. These results also add qualitative nuance to experiences with pharmacy-involved abortion care. Information on patient experiences with pharmacist-involved abortion care is timely as the FDA REMS in-person dispensing requirement for mifepristone has been removed, and as a result at least two major retail pharmacy companies in the US have begun dispensing mifepristone in certain states.^40^

An unexpected finding from this research is that although most participants were satisfied with this overall model of abortion care, gaps in comfort and access persist, especially among adolescents. These limited findings suggest that adolescents experience barriers to care, and although mail-order telemedicine was an opportunity to obtain abortion care, there were distinct unmet needs within this model as well. These obstacles have likely intensified since *Dobbs,* and further research is needed to understand barriers and inform support for adolescents.^41^

### Limitations and strengths

This study has several limitations. The study examines only one provider and abortion regimen. Our findings are not generalisable to the experiences of all people who self-manage their abortion, nor to all those who use misoprostol for abortion. The interviews in this study were conducted 13–16 months after the abortion and participants may have been subject to biases in their recollection of past events.^42^ However, it is also possible that the time elapsed provided sufficient time and space for participants to fully process and reflect on their experiences. Participants had their abortions between May and June 2020 during the COVID-19 pandemic and lockdown period, representing a specific context where individuals’ abortion options were constrained. This likely impacts the generalisability of these findings to other studies on misoprostol or the two service delivery models.

The study’s primary strength is to offer insight into a unique population of people who used misoprostol acquired from a mail-order or retail pharmacy, a novel contribution to the literature that can inform future abortion care models in the US. Since misoprostol-only regimens are not typically offered in US clinics, it is difficult to capture these experiences with facility-based interview recruitment methods. Considering the stigma surrounding abortion and the legal risks of self-managed abortion, the security protocols built into the data collection process may have increased participants’ willingness to share their experiences. This novel dataset offers an opportunity to qualitatively understand the experiences of a hard-to-reach population.

## Conclusion

To conclude, these results contribute to a growing evidence base on the acceptability of multiple abortion service delivery models and the acceptability of the misoprostol-only medication abortion regimen. Understanding the acceptability of misoprostol-only and mail-order and retail pharmacy service delivery is important because medication abortion regimens and abortion care broadly continue to be restricted worldwide. As clinicians, activists and community members continue to pioneer and expand innovative models of abortion care, it is key that these models are evaluated to ensure they are person-centred innovations in abortion care provision and expand reproductive autonomy.

## Footnotes

* We did not collect first or last names, and therefore all names used in the study are pseudonyms.

## Provenance statement

This article was not commissioned and went through external peer review.

## Appendix 1: In-depth interview guide domains and corresponding questions

**Table:**

Domain of acceptability	In-depth interview guide questions
Perceptions of misoprostol	What did you think about the prescription for misoprostol, the medication [the service] prescribed? To what extent have you heard about another process for an abortion with pills, using misoprostol and another medication called mifepristone?
Experiences with the mail-order or retail pharmacy pick-up model	What was your experience with obtaining the prescription? Did you pick up the medications at a pharmacy, or receive the medications via the online pharmacy? [If picked up at the Pharmacy: ] What was your experience with picking up the medication at the pharmacy? Did anyone go with you? How far away was the pharmacy? Did you use the pharmacy you typically use for medicine and other errands, or did you go to another one? What were your interactions like with the pharmacy staff when you were picking up the misoprostol? [If received via online Pharmacy: ] What was your experience with receiving the medicine by mail? What was your experience waiting for the medicine? Were there concerns about receiving medicine by mail? How long did it take to receive the medicines by mail? What were your interactions like with the online pharmacy staff?
Satisfaction	How did you feel about the process you used? Overall, how would you describe your experience using misoprostol for self-managed abortion? How did you feel emotionally during this process? How did you feel physically during this process? What worked well for you, in terms of your abortion experience?

## References

[1] Abortion Care Guideline. World Health Organization; 2022. https://srhr.org/abortioncare/. 35344310

[2] Jayaweera RT, Moseson H, Gerdts C. Misoprostol in the era of COVID-19: a love letter to the original medical abortion pill. Sex Reprod Health Matt. 2020;28(1):1829406–1829406. doi:10.1080/26410397.2020.1829406 PMC7887983 33111643

[3] Coêlho HL, Teixeira AC, Santos AP, et al. Misoprostol and illegal abortion in Fortaleza, Brazil. Lancet (London, England). 1993;341(8855):1261–1263. doi:10.1016/0140-6736(93)91157-h 8098403

[4] Harper CC, Blanchard K, Grossman D, et al. Reducing maternal mortality due to elective abortion: potential impact of misoprostol in low-resource settings. Int J Gynecol Obstetr. 2007;98(1):66–69. doi:10.1016/j.ijgo.2007.03.009 17466303

[5] Kumar R. Misoprostol and the politics of abortion in Sri Lanka. Reprod Health Matters. 2012;20(40):166–174. doi:10.1016/S0968-8080(12)40652-8 23245422

[6] Heidi Moseson P, Ruvani Jayaweera P, Ijeoma Egwuatu B, et al. Effectiveness of self-managed medication abortion with accompaniment support in Argentina and Nigeria (SAFE): a prospective, observational cohort study and non-inferiority analysis with historical controls. Lancet Glob Health. 2022;10(1):e105–e113. doi:10.1016/S2214-109X(21)00461-7 34801131 PMC9359894

[7] Reiss K, Keenan K, Church K, et al. Drug seller provision practices and knowledge of misoprostol in Bangladesh. Int Perspect Sex Reprod Health. 2019;45(1):45–54. doi:10.1363/45e7819 31639080

[8] Foster AM, Messier K, Aslam M, et al. Community-based distribution of misoprostol for early abortion: outcomes from a program in Sindh, Pakistan. Contraception. 2022;109:49–51. doi:10.1016/j.contraception.2022.01.005 35077725

[9] Stillman M, Owolabi O, Fatusi AO, et al. Women’s self-reported experiences using misoprostol obtained from drug sellers: a prospective cohort study in Lagos State, Nigeria. BMJ Open. 2020;10(5):e034670–e034670. doi:10.1136/bmjopen-2019-034670 PMC7223139 32376752

[10] Johnson DM, Michels-Gualtieri M, Gomperts R, et al. Safety and effectiveness of self-managed abortion using misoprostol alone acquired from an online telemedicine service in the United States. Perspect Sex Reprod Health. 2023;55(1):4–11. doi:10.1363/psrh.12219 36744631

[11] Adashi EY, Rajan RS, O'Mahony DP, et al. The next two decades of mifepristone at FDA: history as destiny. Contraception. 2022;109:1–7. doi:10.1016/j.contraception.2022.01.016 35131290

[12] Aiken ARA, Wells ES, Gomperts R, et al. Provision of medications for self-managed abortion before and after the Dobbs v Jackson Women’s Health Organization decision. JAMA : J Am Med Assoc. 2024;331(18):1558–1564. doi:10.1001/jama.2024.4266 PMC10964154 38526865

[13] Institute, G. Interactive map: US abortion policies and access after Roe; n.d. Retrieved May 22, 2024, from https://states.guttmacher.org/policies/.

[14] Updated mifepristone REMS requirements; n.d. Retrieved February 26, 2025, from https://www.acog.org/clinical/clinical-guidance/practice-advisory/articles/2023/01/updated-mifepristone-rems-requirements.

[15] FOOD AND DRUG ADMINISTRATION ET AL. v. ALLIANCE FOR HIPPOCRATIC MEDICINE ET AL.; 2024. https://www.supremecourt.gov/opinions/23pdf/23-235_n7ip.pdf

[16] State of Missouri; State of Kansas’ State of Ohio v. U.S. Food and Drug Administration; Robert M. Califf; 2024. https://storage.courtlistener.com/recap/gov.uscourts.txnd.370067/gov.uscourts.txnd.370067.195.1.pdf

[17] Jayaweera R, Egwuatu I, Nmezi S, et al. Medication abortion safety and effectiveness with misoprostol alone. JAMA Network Open. 2023;6(10):e2340042–e2340042. doi:10.1001/jamanetworkopen.2023.40042 37889485 PMC10611991

[18] Society of Family Planning. Science says: Misoprostol-only is safe and effective; 2023. https://societyfp.org/wp-content/uploads/2023/02/SFP_ScienceSays_misoprostol.pdf.

[19] Raymond EG, Weaver MA, Shochet T, et al. Clinical outcomes of medication abortion using misoprostol-only: a retrospective chart review at an abortion provider organization in the United States. Contraception. 2023;126:110109. doi:10.1016/j.contraception.2023.110109 37390948

[20] Johnson DM, Ramaswamy S, Gomperts R. Experiences with misoprostol-only used for self-managed abortion and acquired from an online or retail pharmacy in the United States. Contraception (Stoneham). 2024;131:110345–110345. doi:10.1016/j.contraception.2023.110345 38049047

[21] Society of Family Planning #WeCount Report April 2022 to June 2024; 2024. doi:10.46621/728122kflzwf

[22] Fine JB, Mayall K, Sepúlveda L. The role of international human rights norms in the liberalization of abortion laws globally. Health Hum Rights. 2017;19(1):69–80. 28630542 PMC5473039

[23] Sekhon M, Cartwright M, Francis JJ. Acceptability of healthcare interventions: an overview of reviews and development of a theoretical framework. BMC Health Serv Res. 2017;17(1):88–88. doi:10.1186/s12913-017-2031-8 28126032 PMC5267473

[24] Levesque J-F, Harris MF, Russell G. Patient-centred access to health care: conceptualising access at the interface of health systems and populations. Int J Equity Health. 2013;12:18. doi:10.1186/1475-9276-12-18 23496984 PMC3610159

[25] O'Brien BC, Harris IB, Beckman TJ, et al. Standards for reporting qualitative research: a synthesis of recommendations. Acad Med. 2014;89(9):1245–1251. doi:10.1097/ACM.0000000000000388. PMID: 24979285. 24979285

[26] Deterding NM, Waters MC. Flexible coding of in-depth interviews: a twenty-first-century approach. Sociol Methods Res. 2021;50(2):708–739. doi:10.1177/0049124118799377

[27] O’Connor C, Joffe H. Intercoder reliability in qualitative research: debates and practical guidelines. Int J Qual Methods. 2020;19. doi:10.1177/1609406919899220

[28] Rennie DL. 3 - The grounded theory method: application of a variant of its procedure of constant comparative analysis to psychotherapy research. In: Fischer CT, editor. Qualitative research methods for psychologists. Elsevier; 2006. p. 59–78. doi:10.1016/B978-012088470-4/50006-5

[29] Huss L, Diaz-Tello F, Samari G. Self-care, criminalized: the criminalization of self-managed abortion from 2000 to 2020. If/When/ How: Lawyering for Reproductive Justice; 2023.

[30] Guttmacher Institute. 2021 is on track to become the most devastating antiabortion state legislative session in decades; 2021. <https://www.guttmacher.org/article/2021/04/2021-track-become-most-devastating-antiabortion-state-legislative-session-decades> [accessed 22 May 2024].

[31] Tousaw E, Moo SNHG, Arnott G, et al. “It is just like having a period with back pain”: exploring women’s experiences with community-based distribution of misoprostol for early abortion on the Thailand–Burma border. Contraception. 2018a;97(2):122–129. doi:10.1016/j.contraception.2017.06.015 28780239

[32] Tang OS, Miao BY, Lee SWH, et al. Pilot study on the use of repeated doses of sublingual misoprostol in termination of pregnancy up to 12 weeks gestation: efficacy and acceptability. Hum Reprod. 2002a;17(3):654–658. doi:10.1093/humrep/17.3.654 11870118

[33] Mitchell EMH, Kwizera A, Usta M, et al. Choosing early pregnancy termination methods in urban Mozambique. Soc Sci Med. 2010;71(1):62–70. doi:10.1016/j.socscimed.2010.03.025 20452107

[34] Madera M, Johnson DM, Broussard K, et al. Experiences seeking, sourcing, and using abortion pills at home in the United States through an online telemedicine service. SSM Qual Res Health. 2022;2:100075. doi:10.1016/j.ssmqr.2022.100075 37503356 PMC10372773

[35] Koenig LR, Ko J, Valladares ES, et al. Patient acceptability of telehealth medication abortion care in the United States, 2021–2022: a cohort study. Am J Public Health. 2024;114(2):241–250. doi:10.2105/AJPH.2023.307437 38237103 PMC10862199

[36] Johnson D, Thompson T-A, Fix L, et al. Perceptions and experiences with two no-test direct-to-patient telehealth medication abortion regimens in the USA: an exploratory study with mifepristone and misoprostol and misoprostol-only regimens. BMJ Publ Health. 2024;2(2). doi:10.1136/bmjph-2023-000808 PMC11816198 40018617

[37] Society of Family Planning. #WeCount report April 2022 to December 2023; 2024. doi:10.46621/970371hxrbsk

[38] Cohen DS, Donley G, Rebouche R. Abortion pills. Stanford Law Rev. 2024;76(2):317–402.

[39] Grossman D, Baba CF, Kaller S, et al. Medication abortion with pharmacist dispensing of mifepristone. Obstet Gynecol. 2021;137(4):613–622. doi:10.1097/AOG.0000000000004312 33706339 PMC7984759

[40] Belluck P. CVS and Walgreens will begin selling abortion pills this month. The New York Times; 2024. https://www.nytimes.com/2024/03/01/health/abortion-pills-cvs-walgreens.html.

[41] Maslowsky J, Lindberg L, Mann ES. Adolescence post-dobbs: a policy-driven research agenda for minor adolescents and abortion. Ann Arbor (MI): Youth Reproductive Equity; 2024. doi:10.7302/22808

[42] Ritchie J. Qualitative research practice. SAGE Publications Ltd; 2023. https://uk.sagepub.com/en-gb/eur/qualitative-research-practice/book237434.

